# Evaluating the implementation of integrated knowledge translation in a multi-country research consortium in Sub-Saharan Africa – a mixed methods comparative case study

**DOI:** 10.1186/s13012-026-01494-3

**Published:** 2026-04-30

**Authors:** Kerstin Sell, Eva Rehfuess, Esther Bayiga-Zziwa, Jimmy Osuret, Lisa Pfadenhauer

**Affiliations:** 1 Institute for Medical Information Processing, Biometry and Epidemiology, Faculty of Medicine, LMU Munich, Elisabeth-Winterhalter-Weg 6, Munich, 81377 Germany; 2 Pettenkofer School of Public Health, Munich, Germany; 3 Department of Disease Control and Environmental Health, School of Public Health, Makerere University, Kampala, Uganda

**Keywords:** Public health, Knowledge translation, Process evaluation, Implementation, Non-communicable diseases, Sub-Sahara Africa, Research co-production, Health research partnership, Evidence-informed decision-making

## Abstract

**Background:**

Integrated knowledge translation (IKT) is an approach facilitating collaboration between researchers and decision-makers towards evidence-informed decision-making. Increasingly evaluated in various contexts, less is known about the implementation process of IKT, including in low- and middle-income countries. The *Collaboration for Evidence-based Healthcare and Public Health in Africa* (CEBHA+) developed, implemented and evaluated an IKT approach across five countries. Here, we examined how the IKT approach was implemented in the African-German multi-country research consortium, investigating project-level context; implementation process, strategy, and outcomes; and exploring intervention core components.

**Methods:**

This process evaluation used a mixed-methods comparative case study design. Following a previously published protocol, the main authors of this paper surveyed and interviewed African CEBHA+ researchers and their partners from policy and practice in 2020/2021 and 2022/2023 and identified relevant IKT-related documents. We drew on our programme theory and implementation science frameworks to undertake qualitative content analysis of interview data and documents. Data was analysed within sites, integrated with descriptively analysed quantitative survey data, and subsequently compared across sites.

**Results:**

We enrolled 36 researchers and 19 decision-makers and analysed 92 IKT-related documents. IKT was implemented at the five sites in Ethiopia, Malawi, Rwanda, South Africa, and Uganda. In our cross-site analysis of fidelity and adaptability of IKT, we identified three core components of the IKT approach: (i) continuous tailored engagement between researchers and decision-makers, (ii) researchers’ commitment to research impact, and (iii) linking to existing KT routines. The context analysis revealed that IKT implementation was facilitated by local KT structures, pre-existing knowledge translation routines and relationships with decision-makers, senior leadership motivation, and funder support including a dedicated budget for IKT activities. Feasibility of IKT implementation was reduced by administrative challenges, overall project complexity, and conflicting priorities.

**Conclusion:**

This research leveraged a unique opportunity to study a systematic IKT approach implemented across sites in five African countries in the context of a large international research consortium. The findings can inform IKT design and implementation in other multi-site and multi-country projects. Particularly, the identified core components can guide adaptation and refinement of IKT in contextually diverse settings, including low- and middle- income countries.

**Supplementary Information:**

The online version contains supplementary material available at 10.1186/s13012-026-01494-3.

Contributions to the literature

- Integrated knowledge translation (IKT) is an established implementation science intervention to strengthen evidence-informed decision-making but less is known about its implementation in low- and middle-income settings.
- Our study reports on the first comprehensive evaluation examining project-level IKT implementation in a multi-country research consortium operating in five African countries.
- Across implementation sites, we identified three generalizable core components that can guide IKT implementation and adaptation in new and diverse contexts, namely (i) continuous tailored engagement between researchers and decision-makers, (ii) researchers’ commitment to research impact, and (iii) linking to existing KT routines.

## Background

Integrated knowledge translation (IKT) is a health research partnership approach aimed at advancing evidence-informed decision-making in public health and healthcare. In IKT, knowledge users, such as policymakers and practitioners, work with researchers throughout ‘the entire research process […] to determine the research questions, deciding on the methodology, being involved in data collection and tools development, interpreting the findings, and helping disseminate the research results.’ [1]. By involving knowledge users who have the power to act on research findings and related recommendations [2], IKT is intended to lead to (i) the production of evidence that is applicable and contextually relevant; and to (ii) increased knowledge user receptiveness and use of research evidence [3]. From an implementation science perspective, IKT is an implementation strategy for evidence-based policy and practice, with the *development of stakeholder interrelationships* [4] at its core. But IKT is also an evidence-based practice itself and as such can be considered an intervention [5].

IKT is implemented widely, in particular in Canada, where it was developed [6, 7], and in Anglophone countries [8]. Fewer examples of IKT implementation exist in African countries, despite a plethora of other knowledge translation (KT) approaches implemented in the region [9]. Evaluations of IKT have delineated *how* the approach works at the researcher-decision-maker interface [10, 11], examined implementation processes [12–15], and assessed how contextual factors influence this interaction and determine ‘success’ in both primary research [10, 11, 16–18] and evidence synthesis [6, 8]. To date, the approach remains more commonly applied in healthcare as opposed to public health contexts, often focused on clinical decision-makers [19].

In the *Collaboration for Evidence-based Healthcare and Public Health in Africa* (CEBHA+), we had the opportunity to study implementation of an IKT approach across a large international research consortium made up of nine academic institutions based in Ethiopia (ET), Germany, Malawi (MW), Rwanda (RW), South Africa (SA), and Uganda (UG). Between 2017 and 2023, research was focused on (i) the prevention and integrated care of non-communicable diseases (NCDs); and (ii) the prevention of road traffic injuries. These focus areas had been determined in a priority-setting exercise with decision-makers and researchers during grant-writing [20]. Alongside the research activities, the consortium implemented an IKT approach. Researchers at Ludwig-Maximilians-Universität (LMU Munich), Germany, and Stellenbosch University, South Africa, led on the development of the approach and guided its implementation; seven academic institutions in the five African countries (‘sites’) implemented the approach. The evaluation of the approach was undertaken by LMU Munich as a non-implementing consortium partner.

In CEBHA+, IKT was an *implementation strategy* for the consortium’s overarching aim to strengthen evidence-based practice and policy with respect to NCD prevention and care. Additionally, we conceptualised the IKT approach as an *intervention* that encompassed a range of highly context- and organisation-sensitive and stakeholder-specific IKT activities that were tailored to the five African sites and that we evaluated to contribute to the science on (I)KT. Previous work has described the CEBHA+ IKT approach [21–23], reported on implementation in South Africa [24], and on the evaluation of IKT outcomes [25].

Despite various challenges encountered while implementing the IKT approach [21], it ‘worked’ and IKT activities were associated with changes among researchers and decision-makers, including building of partnerships, changes in attitudes and knowledge related to evidence use and decision-making, the consideration of evidence for decision-making, and the conduct of contextualised and applicable research [25]. This warranted an in-depth look at implementation of the IKT approach, including project-level implementation, beyond what we initially outlined for our process evaluation [22]. We focused this research on fidelity and adaptability of the IKT approach as well as the project and organisational context with a view to informing the adaptation of such a complex social intervention [26] to other contexts.

### Objectives

Our aim was to evaluate how an IKT approach was implemented in a multi-country research consortium focused on NCDs in Sub-Saharan Africa.

Our specific objectives were to examine

- project-level and organisational-level context,
- the implementation process and strategy, as well as
- implementation outcomes, including exploration of core components of the IKT approach.

## Methods

We undertook a mixed-methods comparative case study, as described in our protocol [22]. Between 3/2020–2/2021 (wave 1) and 9/2022–5/2023 (wave 2) we conducted online surveys, face-to-face and online qualitative interviews and focus group discussions (FGD) with CEBHA+ researchers and their partners from policy and practice; we collected IKT-related documents until June 2023. We employed qualitative content analysis for the analysis of interviews and documents [27], drawing on our programme theory as a deductive coding framework. We analysed quantitative data descriptively and subsequently integrated methods per programme theory category. Methods are described in detail in previously published research [21, 25]. We report on our study using the ASSESS tool (Additional file 1). 

### Deviations from the protocol

This process evaluation initially aimed to ‘shed light on the dose, fidelity and quality of the IKT strategies implemented at each site’ [22]. However, due to inconsistent monitoring it was not feasible to examine IKT activities at individual sites in depth. Moreover, initial evaluation outcomes suggested that a stronger focus on understanding implementation of the overarching IKT approach at project-level was merited, resulting in a changed evaluation scope (Table 1).

**Table 1:** Overview of integrated knowledge translation (IKT) implementation levels

	**IKT approach**	
	Overarching** IKT approach**	Site-specific** IKT activities**
Implementation level	Project-level implementation	Local-level implementation
Context [28]	CEBHA+ consortium, project and organisational context	CEBHA+ institutions and local context, including existing KT structures
Implementation agents [28]	IKT leads at Stellenbosch University and LMU Munich, IKT team, CEBHA+ researchers	CEBHA+ researchers, decision-makers^a^ at each site
Implementation strategies [28]	Workshop, training, IKT team, regular meetings, continuous exchange about IKT	Development, monitoring, and updating of IKT strategies at each site
Implementation process [28, 29]	Exploring IKT fit for CEBHA+, planning and preparing for IKT workshops, IKT team setup	Exploring IKT fit for local context through stakeholder mapping and analysis, gauging decision-maker interest, planning, preparing, and implementing IKT activities
Implementation theory [28]	Not available	Multiple, e.g. [8, 28, 30], as described previously [21]
Implementation outcomes [28, 31, 32]	Fidelity to core components of IKT approach	Fidelity to core components, fidelity to implementing IKT activities as intended

^a^IKT is a research partnership approach focused on decision-makers. However, some CEBHA+ sites involved a broader set of ‘stakeholders’, e.g. academic institutions and non-governmental organisations. Occasionally, we use ‘stakeholder engagement’ terminology to reflect the study participants’ language – recognising that interest-holder may be the more appropriate term [33]

### Implementation-focused data analysis

We conceptualised IKT as a *complex* intervention ‘depend[ant] upon human reaction and reasoning on the part of both the intervention recipients and the individual(s) providing the intervention’ [26]. We thus drew on complexity-informed implementation science frameworks to guide this research (Table 1). We used the *Context and Implementation of Complex Interventions* (CICI) framework [28] to guide the development of our programme theory and evaluation instruments [22], and to provide a structure for the analysis of context and implementation domains. We chose the model for *Exploration, Preparation, Implementation, and Sustainment* (EPIS) for its capacity to examine the implementation process; and for its applicability to IKT given the strong focus on bridging factors, linkages and relationships [29], as previously demonstrated in CEBHA+ [24]. Proctor and colleagues’ (2011, 2013) definitions for implementation strategies [34] and implementation outcomes [31] guided the respective analyses (Tables 1 and 2).

**Table 2:** Implementation science glossary

Implementation process	Defined as ‘…the social processes, through which interventions are operationalised in an organisation or community’ including the ‘tactics and methods used by change leaders’ and ‘corrections, refinements or expansions’ which implementation agents use to successfully implement an intervention [28]. A four-phase model outlines stages of the implementation process, i.e. exploration, adoption/preparation, implementation, sustainment (EPIS) [29].
Implementation strategies	Defined as the ‘methods or techniques used to enhance the adoption, implementation, and sustainability of a clinical program or practice’ ([35], quoted in [34]). These include ‘”top down/bottom up”, “push/pull”, and “carrot/stick” tactics’ [34]. Implementation strategies are tailored to the respective context and may include multiple components and ‘as such may be considered an intervention in its own right’ [28].
Implementation outcomes	Defined as the ‘effects of deliberate and purposive actions to implement new treatments, practices, and services’ [31]. Proctor and colleagues (2011) define acceptability, adoption, appropriateness, cost, feasibility, fidelity, penetration, and sustainability as relevant implementation outcomes.
- Acceptability	Defined as ‘the perception among implementation stakeholders that a given treatment, service, practice, or innovation is agreeable, palatable, or satisfactory’ [31].
- Adoption	Defined as the ‘intention, initial decision, or action to try or employ an innovation or evidence-based practice’ [31].
- Appropriateness	Defined as the ‘perceived fit, relevance, or compatibility of the innovation or evidence-based practice for a given practice setting, provider, or consumer; and/or perceived fit of the innovation to address a particular issue or problem’ [31].
- Cost	Defined as ‘cost impact of an implementation effort’ [31].
- Feasibility	Defined as ‘the extent to which a new treatment, or an innovation, can be successfully used or carried out within a given agency or setting’ ([36], quoted in [31]).
- Fidelity	Defined as the ‘degree to which an intervention was implemented as it was prescribed in the original protocol or as it was intended by the program developers’ [31].
Adaptability	Defined as the degree to which an intervention ‘can be modified, tailored, or refined to fit local context or needs’ [37].
- Core components	Defined as ‘essential and indispensable elements of the intervention itself’ ([38, 39], quoted in [40]), which are ‘responsible for the effectiveness of the intervention’ [41]. According to Medical Research Council guidance for the evaluation of complex interventions, core components are adaptable to enable transportability to different contexts as long as they maintain ‘functional fidelity’ [42, 43]. Their ‘minimally prescribed’ form enhances adoption [43]. We understood core components as ‘capacities and processes to be identified and coached or better rewarded’ as opposed to fixed ‘components to be delivered and counted’ [43].
Mechanisms of impact/change	Mechanisms of change (or impact) describe ‘the causal links between intervention components and outcomes’ as well as the interlinkages between context and the intervention [42].

With respect to the implementation outcome ‘fidelity’, we also examined ‘adaptability’, which describes how an intervention is ‘modified, tailored, or refined to fit local context or needs’ [37], ultimately aiming to identify likely core components of the IKT approach. Core components, essential elements of an intervention, can be ‘unpacked’ when an intervention has been adapted in various contexts [37], which applies to IKT. To achieve this, we iteratively drew on (i) our knowledge as developers of the IKT approach (‘canvassing the designers of the intervention’ [44]), (ii) our extensive evaluation data, in particular qualitative data, (iii) previous publications on IKT in CEBHA+ [20, 21, 23–25, 45], and (iv) discussions among the authors. We identified candidates for core components in this process, reviewed our data to determine whether these components had been implemented as intended, and explored if they had been essential for intervention impact [44].

### Ethics

See Declarations.

## Results

We enrolled 36 researchers and 19 decision-makers in two study waves (2020/2021, 2022/2023) and included 92 IKT-related documents [25] (Table 3).

**Table 3:** Participant characteristics

	**CEBHA+ researchers**		**Decision-makers**	
	**1**^**st**^** wave** 3/2020–2/2021	**2**^**nd**^** wave** 9/2022–5/2023	**1**^**st**^** wave** 3/2020–2/2021	**2**^**nd**^** wave** 9/2022–5/2023
**Total N participants**	25	27	10	11
**Country**				
Ethiopia	2	5	0	2
Malawi	5	6	4	3
Rwanda	6	6	0	4
South Africa	7	7	1	0
Uganda	4	3	5	2
**Gender**				
Female	16	13	1	4
Male	7	13	6	7
Prefer not to say	1	0	0	0
**Professional background**				
Public Health	11	13	1	6
Medicine	10	7	3	1
Epidemiology	2	0	0	0
Other	1	5	3	4
**Institution**				
University	19	18	0	0
Research Institute (not within a university)	5	7	0	1
Government department	0	1	5	8
Regional or local health authority	0	0	1	0
Non-governmental organisation	0	0	0	2
**Overall work experience in the field**				
5 years or less	2	5	1	1
6–10 years	9	3	2	4
11–15 years	7	8	1	4
More than 15 years	5	11	3	2

Below, we report results regarding project context, organisational context, implementation process (i.e. exploration, preparation, implementation, sustainment), implementation strategy, and implementation outcomes (acceptability, adoption, cost, feasibility, fidelity and adaptability). In the context of fidelity and adaptability, we explore core components of the IKT approach.

### Project context

Funder support and interest enabled IKT to be implemented in CEBHA+. The funder had required grant applicants to outline their policy engagement plans and had made funding available for such activities. Throughout the project, the funder expressed great interest in IKT implementation in CEBHA+. This was reflected in their request for the IKT approach to be presented at a network meeting including participants from other African-German research consortia, statements in the external, funder-initiated evaluation, and a continued emphasis on policy engagement in the funder’s subsequent calls for proposals. Due to the funder’s emphasis on policy engagement, all CEBHA+ research tasks included IKT-related deliverables in addition to research-related deliverables.

### Organisational context

IKT activities were implemented in organisational contexts that varied across sites and shaped existing KT routines and structures. KT routines, i.e. the CEBHA+ organisation’s or group’s practice of conducting KT, drew on institutional and personal connections, and organisational reputation; and were linked to wider KT structures at some sites. Organisational hierarchies required particular attention by IKT implementers.

Organisations at four sites had long-standing and strong (South Africa, Uganda) or some established KT routines (Malawi, Rwanda, Table 4, Additional file 2). One particularly strong KT routine was described as follows:

‘So there's a long history of working with policy and engaging around evidence […]. And then more recently, through building relationships with policy makers, […] we now are more at the table informing decisions, making decisions together, forging forward methods. So there's a lot of work happening, prior to CEBHA[+] and around CEBHA[+] in […] knowledge translation.’ (Interview, SA researcher 5)

**Table 4:** Organisational context factors influencing IKT implementation

	**Ethiopia**	**Malawi**	**Rwanda**	**South Africa**	**Uganda**
Existing organisational KT routine		(+)	+	++	++
Organisational reputation				++	++
Organisation embedded in KT structures	(+)	+		++	
Organisational hierarchies	*	++	++	+	++
Inter-personal hierarchies	*	+	+		+

Legend: Organisational context factors mentioned as influencing IKT implementation; ++, +, and (+) indicating the extent of influence at the respective site with ++ denoting a strong influence and (+) denoting very little influence; blank cells: factor was not mentioned as relevant; * insufficient data to assess this factor. More details see Additional file 2

Organisations at three sites reported some (Ethiopia, Malawi) to substantial (South Africa) connections to KT structures, i.e. other organisations, institutions, and networks that were undertaking KT locally and provided a joint platform or opportunities for synergies and exchange. In the absence of existing KT routines at one site, linking to such KT structures enabled IKT implementation:

‘CEBHA[+] at [Armauer Hansen Research Institute] was fortunate that a new "Knowledge Management" directorate had just been started […]. They have many of the same goals as CEBHA[+] IKT initiative and have been helpful in linking our team with government policy advisors.’ (2020/2021 survey, ET researcher)

Inter-organisational hierarchies between research and decision-making institutions influenced IKT efforts:

‘[W]hat we put forward as our agenda […] is to ensure that everything is being aligned to [the NCDs] agenda for the Malawi government. So, that’s our main focus [,…] the agenda for the country. And the [CEBHA+ institution] has got its specific roles in supporting that agenda.’ (Interview, MW stakeholder 4)

Power imbalances amplified organisational hierarchies and required tailoring of IKT activities:

‘We tried before when one of our junior staff would go. But then [decision-makers] wouldn’t value it very much. So then I go there myself or explain to them what we do, how we want them to get involved, and I usually go with my staff […]. Then I can introduce them so that they can continue engaging.’ (Interview, RW researcher 3)

### Implementation process

#### Exploration

The implementation of a research co-production approach was central to the CEBHA+ grant proposal. IKT was later identified as the most suitable intervention for continuing to involve decision-makers and facilitating evidence-informed decision-making. IKT subsequently gained a prominent role in the project, beyond what some researchers had anticipated:

‘And it almost feels as if the IKT part was like an afterthought when planning the project. But it became the centre of the project.’ (Interview, SA researcher 4)

#### Preparation

To operationalise IKT, CEBHA+ researchers at LMU Munich and Stellenbosch University conceived the IKT approach and programme theory drawing on the available literature on IKT (in particular [8, 30]) and team experiences, and developed implementation guidance and a protocol for evaluating IKT [22]. Of note, no pragmatic guidance on setting up IKT in a project was available at the time.

In 2018, CEBHA+ researchers were trained for implementing IKT by LMU and Stellenbosch researchers. In this face-to-face workshop, participants were introduced to IKT, conducted a stakeholder mapping and analysis and subsequently developed tailored ‘IKT strategies’ for the five African sites, using tools developed at Stellenbosch University [46, 47]. IKT strategies outlined the aims, messengers, modes of engagements for prioritised decision-makers, and monitoring indicators [46, 47]. Modes of engagement (‘IKT activities’) included formal and informal meetings, phone or social media contact, training sessions, policy and issue briefs, and policy dialogues [21].

The nine CEBHA+ institutions had different project start dates due to variable length of grant-related administrative processes. This contributed to low attendance at the initial IKT training and to widely varying timelines with respect to IKT implementation.

‘And if you look at the IKT strategy for […] Uganda, we actually had done most of this work before the [IKT] proposal was out.’ (Interview, UG researcher 1)

Ethiopia was the last site to start CEBHA+ work, due to a change in the participating institution after receipt of the grant. Therefore, the majority of the Ethiopian team was introduced to IKT much later and mainly informally:

‘Only to say that the introduction we received to IKT at the beginning of [our work in] CEBHA+ was a helpful starting point […], but I think most of the progress we made was through our own learning processes during and after stakeholder engagement.’ (2022/23 survey, ET researcher)

#### Implementation

A thorough description of the IKT approach is published elsewhere [21] with further details presented below.

Initially, implementation support was dominated by the LMU Munich and Stellenbosch teams’ focus on the development, implementation and management of IKT strategies. Over time, this shifted to a focus on regular meetings of the IKT team and more site-led IKT implementation.

#### Sustainment

We were not able to assess IKT sustainment after the project ended in 2023.

### Implementation strategy

A range of methods were employed to enable the adoption and implementation of the IKT approach. To facilitate IKT adoption, an initial training in IKT was offered in 2018, attended by seventeen researchers (out of approximately 45 CEBHA+ researchers). The training included the drafting of systematic ‘IKT strategies’ to provide a roadmap for implementation. To facilitate IKT implementation, multiple strategies were employed. An IKT team was set up, made up of the individuals who led IKT implementation at each African site and the first and senior author on this publication, who formed a community of practice. The consortium-wide IKT team convened every three months in virtual meetings to discuss IKT activities, in addition to site-specific IKT meetings. An ongoing exchange on IKT was fostered across the wider consortium, e.g. through IKT sessions at consortium meetings and researchers’ participation in the IKT evaluation. Training for KT and KT-related approaches (e.g. issue briefs) was offered through Stellenbosch University on a yearly basis [46–48].

Use of ‘IKT strategies’ to develop, implement, monitor and tailor IKT activities had initially been conceived as a key implementation strategy. However, these were mainly used in South Africa but less so elsewhere after the initial IKT workshop [21] and were thus not a prerequisite to sites implementing IKT activities. IKT strategies were still perceived to offer valuable support to implementers as they made engagements more ‘deliberate’ and helped junior researchers to learn about and plan for IKT (Ethiopia, Uganda).

### Implementation outcomes

#### Acceptability

The acceptability of the IKT approach among CEBHA+ researchers varied over time. Early in the project, some researchers appreciated the potential for relationship-building, insights, and for evidence use, and some voices were enthusiastic about IKT’s potential for population health impact (Additional file 3). In contrast, one researcher described IKT as a concept coming from ‘the outside’ and potentially inappropriate for their context. They further described IKT as a non-novel concept which was given ‘a different name’, referring to their existing KT practice. This was echoed by another researcher:

‘IKT [..], it was, like, something new. […] but then, [..] its activities were not very new to me.’ (Interview, RW researcher 1)

Over time, IKT became widely accepted even among initial sceptics. Partly, this was driven by increasing familiarity with the term and conceptualisation of existing KT routines as IKT.

Formal planning for IKT using ‘IKT strategies’ was, however, less acceptable. Whilst some researchers appreciated the intentionality, others’ comments implied concerns about too much ‘documentation’.

#### Adoption

The IKT approach was widely adopted, as reflected in the initiation of contacts, continuous engagement with decision-makers, and IKT-related outcomes achieved [25]. In Uganda, substantial interaction with decision-makers pre-dated the launch of IKT:

‘So I know that even before the IKT strategy there was involvement, […] not systematic, but there was ongoing engagement with stakeholders.’ (Interview, UG researcher 3)

In contrast, researchers in Malawi were less engaged:

‘No, we don’t [know what’s happening on IKT at our site], but as I say, we’re just doing some very kind of nuts and bolts [research] work. So we haven’t engaged around the knowledge translation stuff.’ (Interview, MW researcher 6)

Feasibility concerns (see below) and in particular lack of acceptability may have contributed to a lack of buy-in from the wider team:

‘[…] we got trained [in IKT]. We came back to train others. I mean we were not experts, okay. So […] in the way we transferred the information […] were not able to convince people, our colleagues to really take it very serious.’ (Interview, MW researcher 3)

#### Cost

While the funder had allowed budgeting for policy engagement, financial resources for IKT (e.g. for staff time, hosting meetings, travel cost reimbursement) were not sufficiently budgeted for at the different sites. This was because the breadth of IKT activities went far beyond those originally conceived in the grant proposal (focussing on policy briefs and policy dialogues). In most cases, teams were able to shift some budget lines to implement the broader set of activities (IKT meeting minutes).

#### Feasibility

Feasibility of implementing the IKT approach was restricted with respect to the available time of researchers and decision-makers, KT skills, staff turnover, and perceived complexity.

At the onset, CEBHA+ was perceived as a complex project by researchers, given the size of the network, the quantity and breadth of research tasks, the range of planned capacity-building activities, and collaboration of institutions from multiple countries, which likely hampered IKT implementation:

‘I found it quite striking at the [initial workshop] that there was […] a lot of confusion about the different research tasks and the working groups. And there was like […] a big box of elements that were not necessarily tied very well together somehow, especially the understanding thereof.’ (Interview, SA researcher 9)

Research was frequently described as taking priority over IKT-related activities, as were administrative tasks, and reporting to the funder. Moreover, the COVID-19 pandemic disrupted work in the middle of the project period and urgent COVID-19-related tasks were prioritised at all sites. At three sites, these led to increased researcher-decision-maker interaction (Malawi, Rwanda, South Africa).

**Fig. 1:**
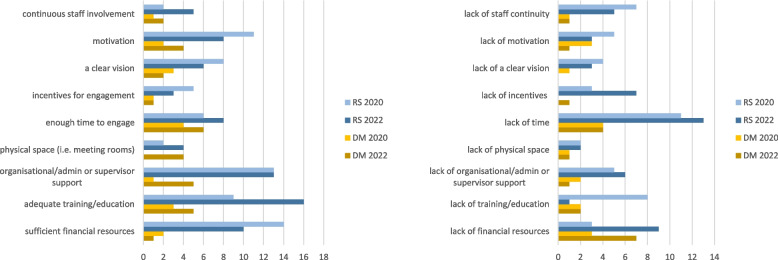
Factors influencing feasibility of IKT implementation. Legend: Factors named as organisational facilitators (left) and barriers (right) to IKT by researchers (RS) and decision-makers (DM) in the online survey. Participants were asked to choose the three most relevant facilitators / barriers from a list.

In our survey, researchers named adequate training, organisational support, motivation and sufficient financial resources as key facilitators for IKT implementation. Lack of time, training, financial resources, and staff continuity were named as key barriers (Fig. 1).

#### Fidelity and adaptability

The CEBHA+ IKT approach foresaw a high degree of tailoring of IKT activities. We thus did not assess whether IKT activities were implemented as initially planned; instead, we examined fidelity to overarching aspects of the IKT approach and identified three core components.

Continuous tailored engagement between researchers and decision-makers constituted one core component of the IKT approach and was implemented with great fidelity [21]. By continuous engagement, we mean ongoing interactions between researchers and decision-makers with varying purpose, intensity and frequency, designed to maintain communication throughout the research process. By tailoring, we mean adaptation of IKT activities by researchers based on deliberate, strategic decisions which take into account the specific context and personalities.

This included consideration of the power imbalance inherent in many relationships with decision-makers. Strategies to tailor IKT activities to high-level decision-makers included, for example, relying on a senior researcher as a messenger for Ministry officials (Rwanda, South Africa, Uganda), supporting the decision-maker’s work, or offering training as a way of building relationships (Malawi, Uganda). Consideration of decision-maker priorities further entailed asking for input into protocols (Rwanda, Ethiopia), changing research questions and scope to meet decision-makers’ needs (South Africa):

‘Those engagements that we have [are] a way of having our finger on the pulse of […] the policy world […] and also which policies are still in the pipeline because, […] we need to know what is coming up so that we can start doing the research so that it can inform [the] policy window.’ (Interview, SA researcher 7)

Lastly, while decisions about decision-maker engagement were made deliberately and strategically, the degree to which this was based on an explicit team-based decision-making process varied.

Commitment to research impact constituted the second core component of the IKT approach. By this, we mean researchers finding inspiration and motivation in the idea of facilitating evidence-informed decision making; and embracing IKT as a strategic approach for improving the relevance and applicability of research outcomes. Where this was embraced, in particular by senior leadership, IKT activities were implemented:

- At the two sites with *strong* existing routines, IKT became embedded in that practice, aligning it with the institutions’ and individuals’ existing commitment to research impact (South Africa, Uganda). Both sites implemented a great range of tailored IKT activities and achieved outcomes linked to IKT.
- At two sites with *less tangible* KT routines, commitment to research impact was – after an initial sense of feeling overwhelmed by IKT as a new concept – strongly embraced by some researchers including senior leadership. This led the Ethiopian team to set up a substantial additional sub-project taking on decision-maker priorities for systematic reviews; and the Rwandan team to supplement its community engagement work with IKT activities.
- At one site with *existing* KT structures but a senior management vacuum, changes in IKT leadership, time and resource constraints, the commitment for research impact was not collectively embraced and IKT activities were more piecemeal and undertaken by individuals (Malawi).

Linking to existing KT routines was identified as the third core component of the IKT approach. Where KT routines pre-existed and the other two core components were adopted (Rwanda, South Africa, Uganda), IKT was integrated into existing practices. In South Africa, this embedding was seamless, given the institutions’ dedication to KT. In Uganda and Rwanda, the existing KT routines were conceptually linked to IKT, subsequently strengthening this engagement and broadening its goals. In Ethiopia, the CEBHA+ team had no existing KT routine. However, identification of and linking with KT structures within and beyond the organisation enabled IKT activities and outcomes. The linking with existing KT routines made IKT more acceptable and meaningful for researchers.

## Discussion

### Summary of findings

We evaluated the process of implementing an IKT approach in the multi-country research consortium CEBHA+ across five countries in Sub-Saharan Africa. Decision-makers were involved continuously throughout the research process, from priority-setting for the consortium until the dissemination of the research findings. Strong funder support including a budget for IKT activities enabled implementation; pre-existing KT routines and relationships with decision-makers, and senior leadership motivation further facilitated implementation. Administrative delays, project complexity, and conflicting priorities presented implementation challenges. Development and monitoring of IKT strategies had initially been conceived as critical implementation strategies but were identified as less relevant. We identified (i) continuous tailored engagement, (ii) commitment to research impact, and (iii) linking to existing KT routines as core components of the IKT approach.

### Contextualizing our findings in the literature

#### Organisational and project context

IKT is applied widely [6], including in African settings [49–51] where it is implemented alongside similar integrated approaches, e.g. knowledge brokering or buddying [52–54], KT platforms [9, 55–58], and rapid response mechanisms [59, 60]. An evidence map of KT strategies used to interact with public health policymakers in African health systems identified 62 studies, with most research from South Africa and Uganda [61]. While no process evaluation of IKT has been conducted in the African region, Edwards and colleagues’ (2019) scoping review assessed organisational context for KT strategies and identified strong institutional links, local champions, and formalised processes for KT as main facilitators; and insufficient capacity, lack of time and resources, and funding constraints as the main barriers. While this in principle mirrors our findings, funding presented less of a barrier as a budget for IKT was available in CEBHA+. Hierarchical power dynamics between health system actors were mentioned as another barrier, albeit rarely [61]. This is echoed in other meta-research examining IKT implementation across 35 IKT case studies in Canada, including seven cases with international collaboration [6]. In this work, Dunn and colleagues (2023) describe ‘balancing power differentials’ as an important facilitator for IKT. In IKT literature, this is often described as more power resting within researchers and academic institutions [62–64]. In contrast, in our study, researchers working with Ministry officials faced the reverse power imbalance requiring deliberate efforts by researchers to address this, which may be specific to working with policymakers.

#### Implementation outcomes

##### Acceptability

IKT was initially met with scepticism by some researchers but eventually became widely accepted, in particular as the approach’s links and similarities with existing ‘stakeholder engagement’ and KT routines became clear. Partly, the ‘socio-technical processes of ‘doing’ this process evaluation’ [65] kept it on the agenda of the research consortium and gradually increased acceptability.

This initial ‘clash’ between a concept established in the international scientific community and local KT routine resonates with other work. Researchers using collaborative research approaches felt some concepts were just ‘repackaging with a new name’ and ‘academic terminology’, preferring approaches that work for their collaboration goals over specific labels [66].

Initial reluctance to adopt the IKT approach perceived as ‘foreign’ is likely further grounded in the experience of African researchers that research agendas in foreign donor-funded projects can have ‘little relevance to local contexts and circumstances’ [67]. In a recent review, 80% of KT-focused studies in African settings were foreign donor-funded [61]. In CEBHA+, the consortium leaders aimed to mitigate this by setting research priorities with African decision-makers [20].

##### Fidelity and adaptability

Adaptation of interventions is ideally undertaken in a structured process [41, 68], and constitutes a defining feature of implementation science – including in low-resource settings [69]. CEBHA+ IKT implementers adapted the IKT approach by (i) foregoing the use of IKT strategies and (ii) resorting to informal monitoring [21]. In light of this, we set out to examine adaptability and identify core components of the IKT approach to inform adaptation in other contexts [42].

While core components are theoretically distinct from mechanisms of impact [70] (Table 2) as the latter are used to describe links with context and outcomes in more depth, and are often examined through realist evaluation [70–72]; they can be difficult to distinguish in evaluation practice [71]. Below, we thus include research evaluating mechanisms of (I)KT as we situate our work in the wider literature, including a recently published realist review on IKT. In this review, Anita Kothari and colleagues (2025) asked ‘What are necessary conditions (context) and key mechanisms that explain the success of IKT in the healthcare sector?’ [71]. They identify infrastructure (including resources and leadership) and role clarity as preconditions for effective partnerships, and power sharing as a precondition for the creation of synergies between partners’ work and goals (Ibid.).

In our work, continuous tailored engagement was perhaps unsurprisingly identified as one of three core components of the IKT approach as it reflects the core characteristics of IKT, as implemented in CEBHA+ [21–24]. The other two core components, commitment to research impact and linking to existing KT routines, are newly identified, complementary and particularly relevant in project-level implementation, as both contribute to acceptability, motivation and buy-in for IKT among researchers.

This first core component, continuous tailored engagement, corresponds to general principles of health research partnerships, identified in large evidence syntheses, that is relationships between researchers and stakeholders [7], effective partnerships [71], their meaningfulness [7], and the nature of interaction [73]. However, we found tailoring to be the distinctive characteristic that made continuous engagement meaningful and effective. Tailoring helped contextualise IKT for individual relationships and for the ‘cultural, political and economic decision-making context’ [67].

At the same time, some decisions about engagements were made implicitly and not through explicit team deliberation, which risks leading to engagement without clear goals. Unstructured interactions have been demonstrated to be less effective in improving evidence use in a review of reviews [73]. The ‘IKT strategies’ initially intended to support implementation may help making engagement aims, messages, and tailored activities explicit.

The comparability of our core components, commitment to research impact and linking to existing KT routines, with other (I)KT evaluations is more limited given our focus on project-level IKT implementation, that is aiming to understand what drives researchers’ adoption of IKT (Table 1).

Commitment to research impact links to Kothari’s (2025) leadership mechanism. Fostering collective commitment requires leadership, which was evident at one site where a leadership vacuum led to limited commitment to research impact and piecemeal IKT. Other realist evaluations have identified further mechanisms which tie into this core component, including researcher motivation [10] and researcher readiness for IKT – a process of researchers overcoming initial scepticism, becoming increasingly interested in KT and subsequently engaging in more KT activities [11]. With leadership, researcher motivation, and researcher readiness all contributing to commitment to research impact we consider this core component as plausible. It represents the introduction of ‘appropriate ideas and opportunities’, a previously described mechanism of impact of IKT ([72, 74], quoted in [26]).

Leadership and resources [71] interface with linking to existing KT routines. This core component entails drawing on existing resources, structures, routines, partnerships, and experiences to enable IKT and to facilitate or maintain effective partnerships. It was key to increasing acceptability and buy-in for IKT, validating existing experience, and managing scarce resources by avoiding duplication of KT efforts. Linking efforts ranged from integrating IKT language and ideas into existing practices to seamless embedding of IKT at the institutions with strong pre-existing KT infrastructure.

Role clarity between researchers and partners, another condition identified in Kothari’s (2025) review did not feature in our work. This is likely due to our focus on project-level implementation.

### Reflexivity

The German and South African CEBHA+ teams led on the development and implementation of the IKT approach and CEBHA+ researchers from the five African countries implemented it at their respective sites. Data were primarily analysed and results drafted by the German team, which carries the inherent risk of adopting a ‘foreign gaze’ [75]. North-South research partnerships are traditionally associated with power asymmetries [76], and the evaluation could be skewed by a paucity of African perspectives. The (African) co-authors of this evaluation had deep contextual knowledge and reviewed results critically, but were involved in IKT implementation, which risks introducing confirmatory bias. These conditions and our positionality may hence have precluded acquiring optimal understanding of some reported processes. The results presented here are however regarded as robust, reporting phenomena observed across sites, time points, and data sources.

### Strengths and limitations

This process evaluation capitalised on a unique opportunity to study IKT implementation across multiple countries and institutions. Our comparative case study design allows for identifying commonalities and differences across contexts, in order to distil generalizable aspects of IKT implementation. While the study design was appropriate for our research questions, a realist evaluation for the development of generalizable mechanisms may have been stronger but was beyond the scope of the project. Realist evaluation is a theory-driven evaluation approach which is intended to delineate how, why, and under which circumstances interventions work [26, 72]. Our examination of core components and realist evaluation share the goal of identifying generalizable components or mechanisms that (can) inform the implementation of an intervention in another setting. Our evaluation is situated between realist evaluation and ‘barriers and facilitators’-focused evaluations, which have been criticised for their limited potential for understanding ‘context and complexity’ [77].

Further limitations arise from the small number of participants from some sites, inconsistent monitoring, and social desirability-bias reflected in some responses, which may have reduced the validity of our findings. Data were collected longitudinally during IKT implementation but analysed retrospectively, which may have introduced additional bias. We were not able to examine the implementation outcomes penetration and sustainability.

Despite these limitations, our study offers important implementation science insights from five African countries, addressing the lack thereof in the field - in which ‘foundations […] continue to be based predominantly on work that is conducted in high-income settings’ ([78], quoted in [69]).

## Conclusion

Our study is the first to examine project-level implementation of IKT in five African countries. We identified continuous tailored engagement, commitment to research impact, and linking to existing KT routines as core components of the IKT approach, which are likely generalizable to other settings. While continuous tailored engagement describes the core IKT work and ensures IKT activities are meaningful, the other two core components are particularly relevant for project-level implementation. Fostering a commitment to research impact can help increase researchers’ motivation for IKT. Linking IKT to existing KT routines and structures ensures contextual relevance. Funders can support IKT implementation with dedicated budgets for research partnerships, which should include some flexible funding to implement activities as needs arise.

## Supplementary Information

Additional file 1: ASSESS reporting guidance.

Additional file 2: Qualitative data supporting organisational context findings.

Additional file 3: Qualitative data supporting implementation outcomes findings. 

## Data Availability

The datasets generated and analysed during the current study are not publicly available due to anonymity concerns but are available from the corresponding author on reasonable request.

## Abbreviations

CEBHA+: Collaboration for Evidence-based Healthcare and Public Health in Africa
FGD: Focus group discussion
ET: Ethiopia
IKT: Integrated knowledge translation
KT: Knowledge translation
LMU: Ludwig-Maximilians Universität München, Germany
MW: Malawi
NCDs: Non-communicable diseases
RW: Rwanda
SA: South Africa
UG: Uganda
