# Correction: Evaluating the implementation of integrated knowledge translation in a multi-country research consortium in Sub-Saharan Africa – a mixed methods comparative case study

**DOI:** 10.1186/s13012-026-01518-y

**Published:** 2026-07-08

**Authors:** Kerstin Sell, Eva Rehfuess, Esther Bayiga-Zziwa, Jimmy Osuret, Lisa Pfadenhauer

**Affiliations:** 1 Institute for Medical Information Processing, Biometry and Epidemiology, Faculty of Medicine, LMU Munich, Elisabeth-Winterhalter-Weg 6, Munich, 81377 Germany; 2 Pettenkofer School of Public Health, Munich, Germany; 3 Department of Disease Control and Environmental Health, School of Public Health, Makerere University, Kampala, Uganda

**Correction: Implementation Science (2026) 21:33**

10.1186/s13012-026-01494-3

In this article, the statement in the Funding information section was incorrectly given as ‘Open Access funding enabled and organized by Projekt DEAL.’ and should have read ‘Open Access funding enabled and organized by Projekt DEAL. CEBHA+ was funded by the German Federal Ministry of Education and Research (Bundesministerium für Bildung und Forschung) as part of the “Research Networks for Health Innovation in Sub Saharan Africa” funding initiative. Grant nos: 01KA1608, 01KA2023, 01KA2111A. The funder was not involved in the study design, collection, analysis, interpretation of data, the writing of this article or the decision to submit for publication’.

The attribution of the CEBHA+ Integrated Knowledge Translation (IKT) Team members (Ann R. Akiteng, Firaol M. Ayele, Bonny E. Balugaba, Gertrude Chapotera, Peter Delobelle, Kiya Kedir, Suzgika Lakudzala, Naomi S. Levitt, Joerg J. Meerpohl, Talitha Mpando, Jean B. Niyibizi, Seleman Ntawuyirushintege, Anke Rohwer and Ingrid Toews) was incorrect in the original publication and has now been corrected in all versions of the article. 

The original article has been corrected.

